# Crebanine induces ROS-dependent apoptosis in human hepatocellular carcinoma cells *via* the AKT/FoxO3a signaling pathway

**DOI:** 10.3389/fphar.2023.1069093

**Published:** 2023-02-16

**Authors:** Jiajie Tan, Yuling Xiang, Yuanguo Xiong, Yaoyuan Zhang, Boyang Qiao, Hong Zhang

**Affiliations:** ^1^ Department of Pharmacy, Renmin Hospital of Wuhan University, Wuhan, Hubei, China; ^2^ School of Pharmaceutical Sciences, Wuhan University, Wuhan, Hubei, China

**Keywords:** hepatocellular carcinoma, crebanine, reactive oxygen species, Akt, FoxO3a, apoptosis

## Abstract

**Background:** Hepatocellular carcinoma (HCC), as an aggressive cancer with a high mortality rate, needs high-efficiency and low-toxicity drug therapy. Natural products have great potential as candidate lead compounds for the development of new HCC drugs. Crebanine is an isoquinoline alkaloid derived from Stephania with various potential pharmacological effects such as anti-cancer. However, the molecular mechanism underlying crebanine-induced liver cancer cells apoptosis has not been reported. Here, we investigated the effect of crebanine on HCC and identified a potential mechanism of action.

**Methods:** In this paper, we intend to detect the toxic effects of crebanine on hepatocellular carcinoma HepG2 cells through a series of *in vitro* experiments, including detecting the effects of crebanine on the proliferation of HepG2 cells using the CCK8 method and plate cloning assay, observing the growth status and morphological changes of crebanine on HepG2 cells by inverted microscopy; and using the Transwell method to determine the the effect of crebanine on the migration and invasion ability of HepG2 cells; using Hoechst 33258 assay to stain cancer cells, thus observing the effect of crebanine on the morphology of HepG2 apoptotic cells, and detecting the apoptotic state and level of HepG2 cells by flow cytometry; using ROS kit and JC-1 assay kit to detect the changes of reactive oxygen species and mitochondrial membrane potential of HepG2 The immunofluorescence assay was taken to verify whether crebanine had an effect on the expression of p-FoxO3a in cancer cells; the Wetern blot assay was also used to examine the effect of crebanine on proteins related to the mitochondrial apoptotic pathway and its effect on the regulation of the relative protein expression of AKT/FoxO3a axis; after this, NAC and AKT inhibitor LY294002 were used to cells were pretreated with NAC and AKT inhibitor LY294002, respectively, in order to further validate the inhibitory effect of crebanine.

**Results:** It was shown that crebanine effectively inhibited the growth and capacity of HepG2 cells migration and invasion in a dose-dependent manner. Furthermore, the effect of crebanine on the morphology of HepG2 cells was observed through microscopy. Meanwhile, crebanine induced apoptosis by causing reactive oxygen species (ROS) burst and mitochondrial membrane potential (MMP) disrupt. We found that crebanine could down-regulate Bcl-2 and up-regulate Bax, cleaved-PARP, cleaved-caspase-3 and cleaved-caspase-9, but these effects were overturned by ROS inhibitor N-acetylcysteine (NAC). Crebanine also down-regulated p-AKT and p-FoxO3a, and the PI3K inhibitor LY294002 significantly enhances this effect. We also found that the expression of AKT/FoxO3a signaling pathway was ROS-dependent. As shown by Western blots, NAC could partially attenuate the inhibitory effect of crebanine on AKT and FoxO3a phosphorylation.

**Conclusion:** Based on our results, our results suggest that crebanine, as a compound with potential anticancer activity, has significant cytotoxic effects on hepatocellular carcinoma,and it likely induces apoptosis *via* ROS in the mitochondrial pathway and simultaneously affects the biological function of HCC *via* the ROS-AKT-FoxO3a signaling axis.

## Introduction

In the world, hepatocellular carcinoma (HCC) is one of the most common malignancies (Sung et al., 2021). Despite the fact that there has been some advancement in the therapy of liver cancer lately, radiofrequency ablation, liver transplantation, and other methods still has many limitations and side effects (Kudo, 2020). Although many other active treatments are available and many new chemotherapy drugs have been developed, the marketed drugs are associated with increased patient mortality due to long courses of treatment, complex compatibility, multiple drug interactions, and high toxicity (Sonbol et al., 2020). Therefore, current treatments for liver cancer still have safety and efficacy limitations. In the past few decades, accumulating evidence has suggested the therapeutic potential of traditional Chinese medicine and natural products as safer alternatives for the treatment of liver cancer (Ma et al., 2019; Xiang et al., 2019). Many active ingredients extracted from traditional Chinese medicine can reduce adverse reactions or drug toxicity caused by surgical treatment and drug therapy, showing unique therapeutic effects as primary treatment and as adjuvant therapy (Luo et al., 2019). These natural active ingredients not only relieve patients’ cancer symptoms, but also improve their quality of life and long-term survival (Wang et al., 2012).

Crebanine, as an monomeric compound, has potential pharmacological effects on arrhythmia, Alzheimer disease, and tumors. Studies have also found multiple therapeutic targets of crebanine (Makarasen et al., 2011; Rojsanga et al., 2012; Yang et al., 2013; Xiao-Shan et al., 2014; Wang et al., 2016). Regarding the oncogenic effects of crebanine, According to earlier research, crebanine inhibited the invasion and migration of TNF-α-stimulated A549 cells and decreased the expression of multiple invasion and migration-related factors such as MMP9, uPA, uPAR, and ICAM1 (Yodkeeree et al., 2014). In addition, Wongsirisin et al. found that crebanine acting on HL-60 cells mediated cell cycle arrest in G0/G1 phase and downregulated cycle-related proteins cyclins A and cyclins D. It also induced apoptosis-related proteins such as PARP, caspase-3, caspase-9, and caspase-8 by inducing cyclins A and cyclins D expression, regulating apoptosis through endogenous and exogenous pathways (Wongsirisin et al., 2012). However, so far, no study has investigated the therapeutic effect of crebanine on HCC cells.

Research has shown that an overabundance of reactive oxygen species (ROS) in cancer cells can cause the production of excessive intracellular free radicals as well as dysfunction of the antioxidant system. This inhibits cell proliferation, induces DNA damage, autophagy, and disrupts intracellular mitochondrial homeostasis (Idelchik et al., 2017; Moloney and Cotter, 2018). The mitochondrial dysfunction causes abnormal expression of anti-apoptotic or pro-apoptotic genes such as Bax, Bim, Bak, Bcl-2 and PARP and stimulates caspase cascade reaction, which ultimately results in apoptosis (Chiu et al., 2020). Apoptosis is the most common way of death of cancer cells after abnormal attack, and among the exogenous and endogenous apoptosis pathways, apoptosis induced by mitochondrial pathway is the most important one (Wang L. et al., 2017).

One of the most important pathways in cancer signaling cascades is the PI3K/Akt pathway. It has been shown to perform a different function in cell proliferation, differentiation, metabolism, and invasion and apoptosis (Liang et al., 2020; Habrowska-Górczyńska et al., 2021). The PI3K/Akt pathway is regulated by upstream targets such as PTEN and it influences the expression of downstream targets such as GSK3-β, FoxO3a, β-catenin, p21, p27, and Mdm2 (Fresno et al., 2004; Nogueira et al., 2008; Patra et al., 2021). Among them, FoxO3a is a member of the Forkhead box O family of transcription factors, which is often stimulated by PI3K/Akt signaling. When in an excited state, FoxO3a controls the regulation of several genes related to cancer, cellular senescence, differentiation, metabolism, and redox dynamic balance (Koundouros and Poulogiannis, 2018; Li et al., 2018).

In this study, we hypothesized that crebanine not only inhibits the proliferation, migration, and invasion of hepatocellular carcinoma, but it can also induce apoptosis through the mitochondrial pathway by activating ROS. Our experimental results confirmed this hypothesis. Notably, we also found for the first time that crebanine-mediated ROS generation is involved in the inhibition of PI3K/AKT/FoxO3a signaling pathway in HCC.

## Materials and methods

### Chemicals and materials

Crebanine with purity above 97% was provided by Chengdu pureChem-standard Corp. (Chengdu, China); Fetal bovine serum (FBS) and dulbecco’s modified Eagle’s medium (DMEM) were obtained from Gibco (Thermo Fisher Scientific, Inc. United States); LY294002 (PI3K Inhibitor) was purchased from MedChemExpress (NJ, United States); N-acetylcysteine (NAC) was obtained from Sigma Chemical (St. Louis, MO, United States); Hoechst 32258 was obtained from Wuhan Servicebio Biotechnology Co., Ltd. (Wuhan, China); CCK-8 kit was purchased from Biosharp (Anhui, China); the ROS assay kit and JC-1 kit were obtained from Beyotime Biotechnology (Shanghai, China); Superoxide Dismutase (SOD) assay kit、Malondialdehyde (MDA) assay kit、Glutathione Peroxidase (GSH-PX) assay kit were purchased from Nanjing Jiancheng bioengineering research institute (Nanjing, China); Annexin V/PI apoptosis detection kit was obtained from BD Biosciences (NY, United States); the monoclonal antibody against Bax, caspase-3, Bcl-2, AKT, p-AKT, FoxO3a, β-actin and horseradish peroxidase (HRP)-conjugated secondary antibodies were obtained from Cell Signaling Technology (CST, United States); the monoclonal antibody against caspase-9, p-FoxO3a (Ser253) were obtained from Proteintech (Rosmont, IL, United States).

### Cell culture

The Shanghai Institute of Biochemistry and Cell Biology (Chinese Academy of Sciences, Shanghai, China) provided HepG2 cells. Growth of the cells was carried out in DMEM containing 10% FBS, 1% penicillin-streptomycin (100 U/mL penicillin and 100 U/mL streptomycin), and 37°C in humidified 5% CO_2_ incubator (Thermo Forma Electron Co, Marietta, OH, United States). Cells were subcultured with fresh media at a frequency of 1:4 every two to 3 days.

### Cell viability assay

Briefly, about 1 × 10^4^ cells/wells were plated into 96-well plates and exposed to crebanine (35, 70, 105, 140, 175, and 280 µM) for 24, 48, 72 h in a 37°C incubator. The cells were cultured in 10 µl of CCK-8 solution for 1 h. Optical density was determined using a microplate reader at a wavelength of 490 nm after incubation. The IC50 values were determined by plotting linear regression curves.

### Colony formation assay

About 4 × 10^3^ cells/well were seeded in 6-well plate overnight. Cells were exposed to crebanine treatment (0, 35, 70, 105, 140, 175, and 280 µM) for 24 h, and then the cells were grown for 14 days with the media being switched out every 3 days. On the 14th day, cells were fixed with 4% paraformaldehyde for 15 min and then stained with crystal violet dye for 25 min. Then, phosphate-buffered saline (PBS) was used to gently wash each well before allowing it to air dry. Colony numbers were then calculated using Image J software (National Institutes of Health, United States).

### Morphological assessment assay

HepG2 cells were seeded into 6-well plates at a density of 6 × 10^5^/well. After overnight incubation, cells were treated with crebanine for 24 h. Then, changes in cell morphology were imaged under an inverted phase-contrast microscope (Olympus, Hamburg, Germany).

### Cell migration and invasion assays

Using 24-well transwell chambers with or without 50 µL of matrigel, cell migration and invasive ability were assessed based on the number of cells passing through the chambers. HepG2 cells were trypsinized after crebanine treatment, and about 5 × 10^4^ cells/well were placed in the upper chamber of the transwell and 0.5 mL of complete medium in the lower chamber before being placed in a 37°C incubator. After 24 h, to remove upper chamber cells, gently wash the chamber twice with PBS using a sterile cotton swab. After that, the cells were fixed with paraformaldehyde, stained with 0.1% crystal violet dye for 20 min, and examined with an inverted phase contrast microscope.

### Hoechst 33258 staining

Application of hoechst 33258 staining to observe the morphology of apoptosis. Briefly, 1 × 10^5^ cells per well were cultured in 6-well plates and then treated with different concentrations of crebanine for 24 h. Subsequently, cells were washed three times with PBS at room temperature, and being fixed with 4% paraformaldehyde for 20 min and stained with 5 μg/mL hoechst 33258 for 30 min. Morphological changes of apoptotic cells were photographed using an inverted phase-contrast microscope.

### Apoptosis detection

Annexin V- FITC/PI staining kit was used to measure apoptotic cells in this experiment. In 6-well plates, about 6 × 10^5^ cells/well were planted and incubated overnight, followed by a 24 h crebanine treatment. Subsequently, the cells were collected by centrifugation, gently washed twice with PBS, and stained with annexin V-FITC/PI for 25 min. Subsequently, the frequency of apoptotic cells was determined using a flow cytometer (CytoFLEX, Backman Counter, CA, United States) under the manufacturer’s instructions.

### Determination of cellular ROS

A fluorescent probe DCFH-DA was used in fluorescence microscopy and flow cytometry to measure changes in intracellular ROS level. About 6 × 10^5^ cells/well were seeded in 6-well plate overnight and then treated with crebanine for 24 h. Subsequently, the cells were collected by centrifugation, suspended in PBS, and loaded with 20 µM DCFH-DA at 37°C for 30 min. Following fluorochrome incubation, cells were washed twice with PBS and analyzed immediately with a flow cytometer using a FL-1 filter with an excitation wavelength of 480 nm.

### Oxidative stress-related molecular assays

Superoxide dismutase (SOD), glutathione (GSH-PX) and malondialdehyde (MDA) assay kits were used to detect intracellular levels of SOD, GSH-PX and MDA, respectively. In 6-well plates, about 6 × 10^5^ cells/well were planted and incubated overnight, followed by a 24 h crebanine treatment, then washed twice with ice-cold PBS and the supernatant was collected by centrifugation for 4 min, then the kit instructions were followed and finally detected on a microplate reader.

### Assessment of mitochondrial membrane potential

JC-1 assay kit was used to detect variation in mitochondrial membrane potential (MMP). A 6-well plate was seeded with about 5 × 10^5^ cells/well, and it was left incubating overnight. The cells were then exposed to crebanine at various doses for 24 h. Following that, cells were obtained by centrifugation, suspended in PBS, and dyed with JC-1 for 20 min at 37°C in accordance with the manufacturer’s instructions. The cells were then collected and given two rinses in JC-1 staining buffer (1×). A flow cytometer was used to immediately analyze stained cells.

### Immunofluorescence

Cells in 6-well plate were washed 3 times with PBS and fixed with 4% paraformaldehyde for 25 min; then, samples were treated in 0.2% Triton X-100 for 15 min and closed with PBS containing 2% BSA for 30 min at room temperature. After that, cells were incubated with primary antibody overnight at 4°C, followed by incubation with FITC-coupled secondary antibody for 2 h at room temperature. Cells were washed three times with PBS and fixed with PBS containing DAPI for 15 min. Cells were visualized under a fluorescent microscope.

### Western blot

In 6-well plates, about 6 × 10^6^ cells were cultivated per well, and they were incubated at 37°C overnight. Crebanine was applied to the cells for 24 h. The cells were then homogenized and dissolved in RIPA buffer in the presence of a protease inhibitor to extract cell proteins. Protein concentrations were measured using the BCA protein determination kit. Samples were separated on 10%–12% sodium dodecyl sulfate-polyacrylamide gel electrophoresis followed by electro-transfer onto PVDF membranes. The primary antibodies of caspase-3 (1:1000), caspase-9 (1:1000), PARP (1:1000), AKT (1:2000), p-AKT (1:1500), FoxO3a (1:1000), p-FoxO3a (Ser253) (1:1000), and β-actin (1:4000)—were then incubated on the PVDF membranes for at least an overnight period at 4°C. Following three 10-min TBST washes, the membranes were incubated for 2 h at room temperature with diluted horseradish peroxidase (HRP)-conjugated secondary antibodies (1:10,000). The membranes were detected using the Odyssey Dual Color Infrared Laser Imaging System (LI-COR, United States) after three TBST washes lasting 10 min each. The outcomes were quantified with Image J software.

### Statistical analysis

Data from at least three independent experiments are presented as the mean ± SD. All data were analyzed by the SPSS 17.0 software, Changes over time and differences between treatment and control groups were analyzed using a one-way analysis of variance (ANOVA) followed by the Tukey’s multiple comparison post-hoc test. If the *p*-value was less than 0.05, the difference was considered statistically significant.

## Results

### Crebanine suppresses cell growth in HepG2 cells

**FIGURE 1:**
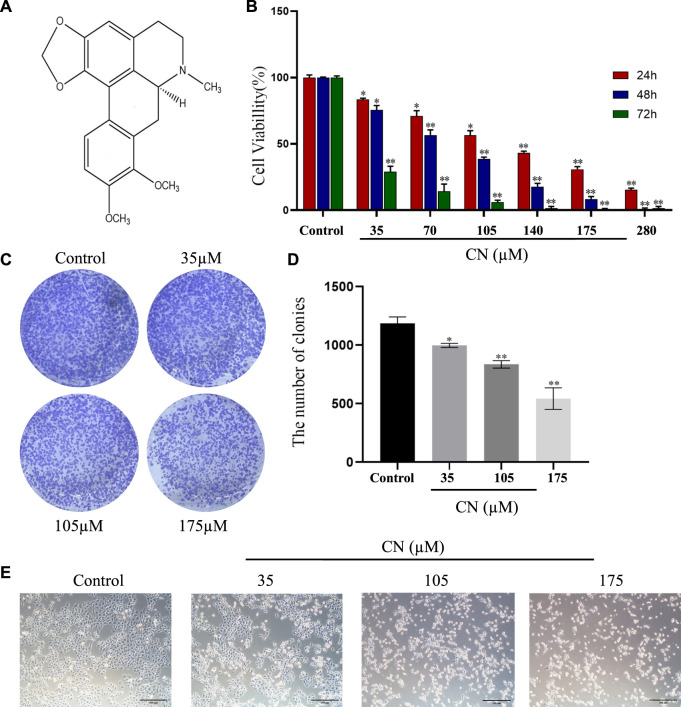
Crebanine promotes growth inhibition in HepG2 cells. **(A)** Structure of crebanine. **(B)** Cell viability of HepG2 cells after crebanine treatment for 24–72 h was detected by CCK8 assay (*n* = 6 for each group, one-way ANOVA with Tukey’s *post hoc* test, *p < 0.05, **p < 0.01, ***p < 0.001 vs. control). **(C)** Colony formation assay of HepG2 cells treated with indicated concentrations of crebanine for 24 h (*n* = 3 for each group). **(D)** Quantitative analysis of colony formation assay (*n* = 4 for each group, one-way ANOVA with Dunnett *post hoc* test, *p < 0.05, **p < 0.01, ***p < 0.001 vs. control). **(E)** Morphology of HepG2 cells following treatment with indicated concentrations of crebanine (*n* = 3 for each group). All data are expressed as mean ± SD.

Our study investigated the cytotoxicity of crebanine on HepG2 cells by treating them with varying concentrations (0, 35, 70, 105, 140, 175, and 280 µM) over 24–72 h. We analyzed the cytotoxicity of crebanine using CCK-8 assays. As shown in Figure 1B, HepG2 cells treated with crebanine showed a sharp decline in viability in a dose- and time-dependent manner compared with the control group. In addition, the IC50 value at 24, 48, and 72 h for cytotoxicity on HepG2 cells was 111.77 ± 5.40 µM, 65.07 ± 0.35 µM, 23.68 ± 2.04 µM respectively. These values showed that HepG2 cells were sensitive to crebanine treatment. Therefore, we chose 35, 105, and 175 µM of crebanine as the experimental group in our subsequent experiments. To determine population dependence and long-term proliferation capacity, cells were incubated with crebanine at concentrations of 35, 105, and 175 µM for 14 days before colony formation. In Figures 1C,D, crebanine significantly reduced HepG2 cells’ ability to form clones, indicating that it inhibited cancer cell proliferation over time and dose-dependently. Subsequently, the impact of crebanine on cell growth was then assessed by examining morphological alterations in HepG2 cells with an inverted microscope. As shown in Figure 1E, crebanine induced significant morphological changes in cancer cells. In the treatment groups, cell bodies generally became smaller and rounder than those of the control group; additionally, significant cellular debris appeared in the culture media, and cell count dropped. From these results, we can konw that crebanine inhibited both proliferation and death of cells.

### Crebanine blocking migration and invasion in HepG2 cells

**FIGURE 2:**
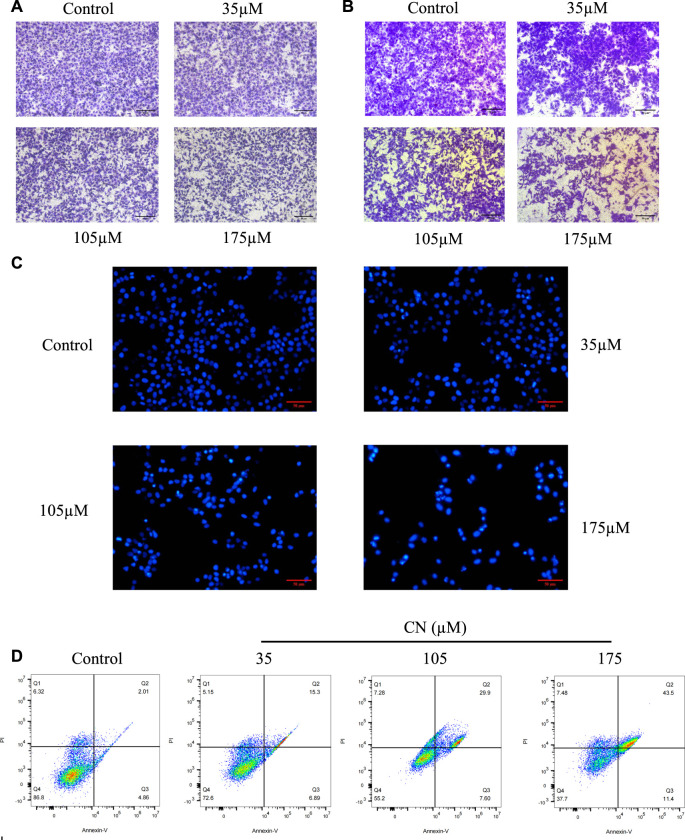
Crebanine inhibits HepG2 cells migration, invasion and promotes apoptosis. HepG2 cells were incubated with crebanine for 24 h; cell migration **(A)** and invasion **(B)** were analyzed using the transwell assays (*n* = 3 for each group). **(C)** Apoptotic morphological changes were evaluated by fluorescent microscopy using Hoechst 33258 staining (*n* = 3 for each group). **(D)** HepG2 cells treated with crebanine were stained with Annexin V-FITC/PI and analyzed by flow cytometry (*n* = 3 for each group).

We used transwell assays to examine crebanine’s impact on HepG2 cells’ capacity for invasion and migration. The findings of treating the cells for 24 h with various doses of crebanine showed that it considerably reduced the ability of HepG2 cells to invade and migrate, and higher crebanine concentrations had stronger inhibitory effects (Figures 2A,B). This finding suggested that crebanine suppressed the migration and invasion of HepG2 cells.

### Crebanine induces apoptosis through activates ROS in HepG2 cells

To determine whether crebanine’s cytotoxicity is due to apoptotic events, we first used Hoechst 33258 to observe nuclear morphological changes during apoptosis. As shown in Figure 2C, cancer cells in the control group were in a good condition-only slightly stained, whereas HepG2 cells in the crebanine-treated group showed a rapid decrease in viability, obvious cell shrinkage, chromatin concentration, and appearance of both fluorescent nuclear and apoptotic fragments. In other words, crebanine causes apoptosis in a dose-dependent way. In addition, we used the flow cytometry to identify a specific apoptosis rate. As can be seen in Figure 2D, crebanine treatment considerably increased the apoptosis rate of cancer cells compared to the control group, with the maximum apoptosis rate of 43.5% occurring in cells that received the highest dose of crebanine. This result further demonstrated the pro-apoptotic effect of crebanine.

**FIGURE 3:**
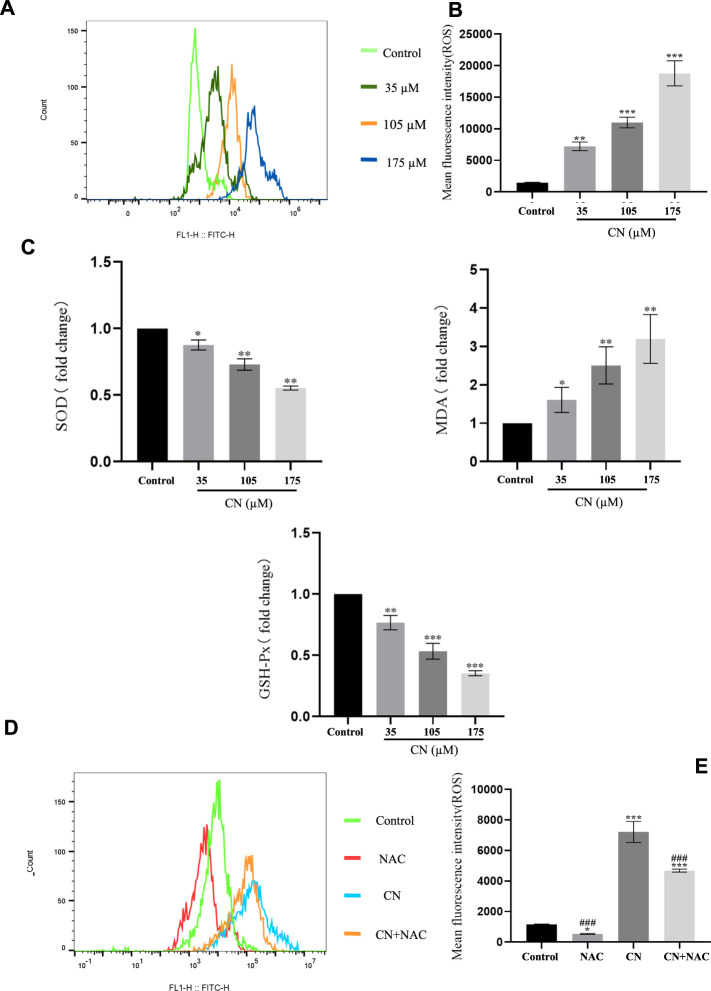
Crebanine induces apoptosis in HepG2 cells is depending on ROS activation. Flow cytometry analysis were performed to determine the intracellular ROS levels in HepG2 cells with **(A)** or without **(D)** 4 mM NAC treatment for 1 h prior to treatment with 105 µM crebanine for 24 h (*n* = 3 for each group). **(B)** and **(E)** Mean fluorescence intensity of ROS (*n* = 3 for each group, one-way ANOVA with Tukey’s *post hoc* test, *p < 0.05, **p < 0.01, ***p < 0.001 vs. control). **(C)** Changes in SOD, MDA and GSH-PX after oxidative stress caused by CR (*n* = 3 for each group, one-way ANOVA with Tukey’s *post hoc* test, *p < 0.05, **p < 0.01, ***p < 0.001 vs. control). All data are expressed as mean ± SD.

In addition, to understand whether crebanine-induced apoptosis was due to excessive production of ROS in HepG2 cells, we detected intracellular ROS production using 2′,7′-dichlorodiacetate fluorescein (DCFH-DA) staining after drug administration. Based on the experimental results in Figures 3A,B, crebanine caused an increase in the level of ROS in hepatocellular carcinoma cells. In addition, SOD, GSH, and MDA changed with the level of oxidative stress, therefore, detecting their fluctuations was beneficial for us to better determine the effect of kebanine on cancer cells. According to the experimental results as shown in Figure 3C, we found that as the concentration of crebanine administration increased, while stimulating the production of ROS, the production of MDA also gradually increased, while SOD and GSH decreased accordingly, and they, as important compounds for maintaining the intracellular redox state, also proved the burst of oxidative stress caused by crebanine from the side.

**FIGURE 4:**
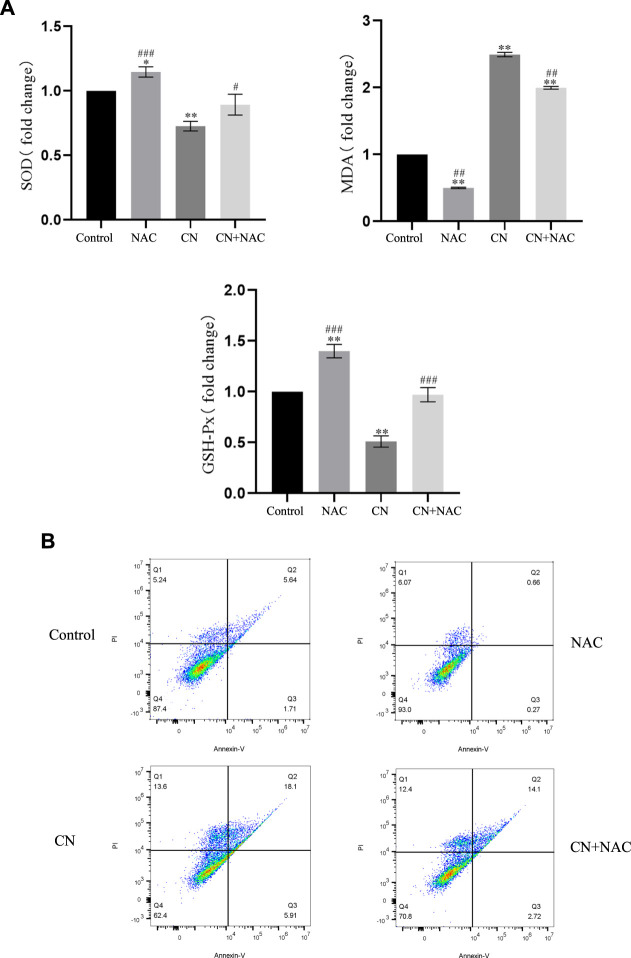
Crebanine induces apoptosis in HepG2 cells *via* the mitochondrial apoptotic pathway. **(A)** Pre-treatment with NAC before giving CN and re-detection of SOD, MDA and GSH-PX changes afterwards (*n* = 3 for each group, one-way ANOVA with Tukey’s *post hoc* test, *p < 0.05, **p < 0.01, ***p < 0.001 vs. control). **(B)** After NAC pretreatment was carried out for 1 h, cells were treated with indicated concentrations of crebanine for 24 h, effect of crebanine on HepG2 cells death as assessed by Annexin V/PI staining (*n* = 3 for each group). All data are expressed as mean ± SD.

Additionally, using the ROS inhibitor NAC substantially reduced the impact of increasing ROS production (4 mM) (Figures 3D,E), Also, the results of Figure 4A suggest that NAC as an anti-oxidative stress compound can modify the effects of SOD, MDA, and GSH on redox status to some extent. Also, the ability of crebanine to induce apoptosis in cancer cells was significantly reduced when we pretreated hepatocellular carcinoma cells with NAC (Figure 4B), confirming that crebanine could induce apoptosis by activating oxidative stress.

### Crebanine indues mitochondrial pathways-related apoptosis through ROS upregulation

**FIGURE 5:**
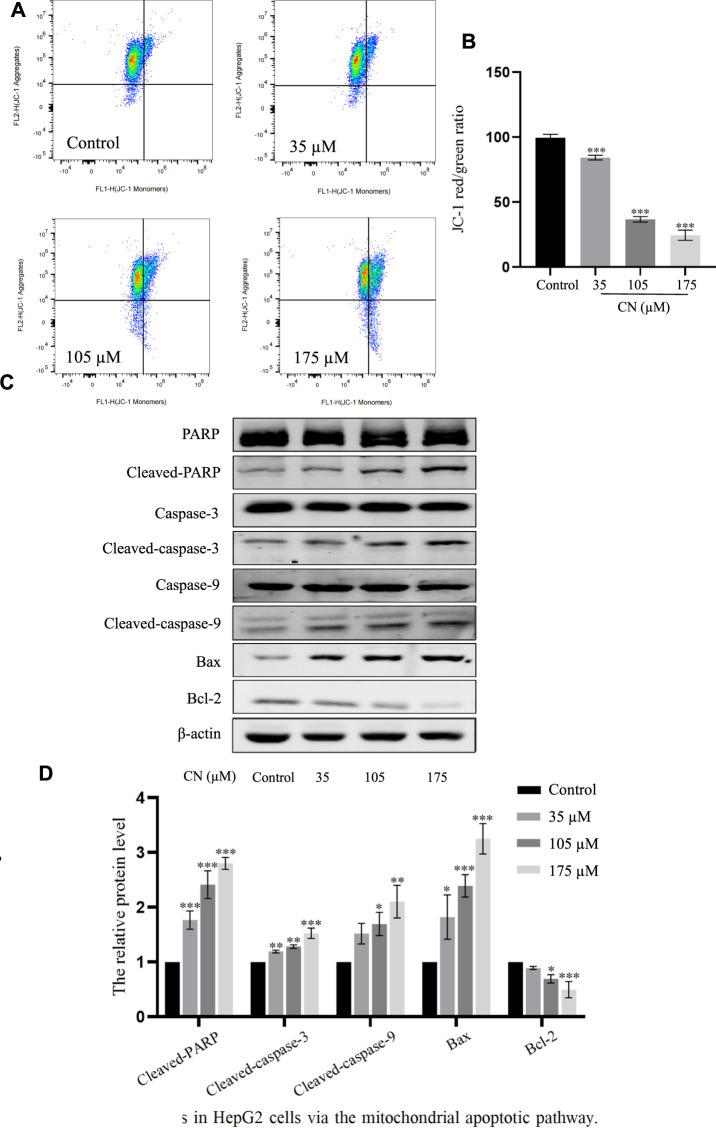
Crebanine induces apoptosis in HepG2 cells *via* the mitochondrial apoptotic pathway. **(A)** JC-1 staining and flow cytometry were performed to evaluate the effect of crebanine on mitochondrial membrane potential (Δψm) in HepG2 cells (*n* = 3 for each group). **(B)** The JC-1 red/green rate for HepG2 cells (*n* = 3 for each group, one-way ANOVA with Tukey’s *post hoc* test, ***p < 0.001 vs. control). **(C, D)** HepG2 cells were treated with various concentrations of crebanine for 24 h, western blot was used to determined the expressions of cleaved PARP, caspase-3, -9, Bcl-2 and Bax (*n* = 3 for each group, one-way ANOVA with Tukey’s *post hoc* test, *p < 0.05, **p < 0.01, ***p < 0.001 vs. control). All data are expressed as mean ± SD.

It has been reported that elevated ROS levels can induce apoptosis through a series of downstream pathways. Mitochondrial dysfunction is one of the major effects of ROS production, which first leads to a decrease in membrane potential, and then induces abnormal expression of apoptosis-related proteins such as PARP, Caspase-3, Caspase-9, Bax, Bcl-2 (Plackova et al., 2016). Therefore, to assess whether crebanine caused mitochondrial alterations in hepatocellular carcinoma cells, we examined the mitochondrial membrane potential. After administration of crebanine at different concentrations, flow cytometry results for this detection are shown in Figures 5A,B, the mitochondrial membrane potential of HepG2 cells treated with crebanine decreased in a concentration-dependent manner, suggesting the involvement of mitochondrial apoptosis pathway in crebanine-induced apoptosis. Western blot results in Figure 5C and the protein relative expression in Figure 5D showed that the levels of Bax, cleaved-PARP, cleaved-caspase-3 and cleaved-caspase-9 were significantly increased after crebanine treatment, whereas the expression level of Bcl-2 was decreased.

**FIGURE 6:**
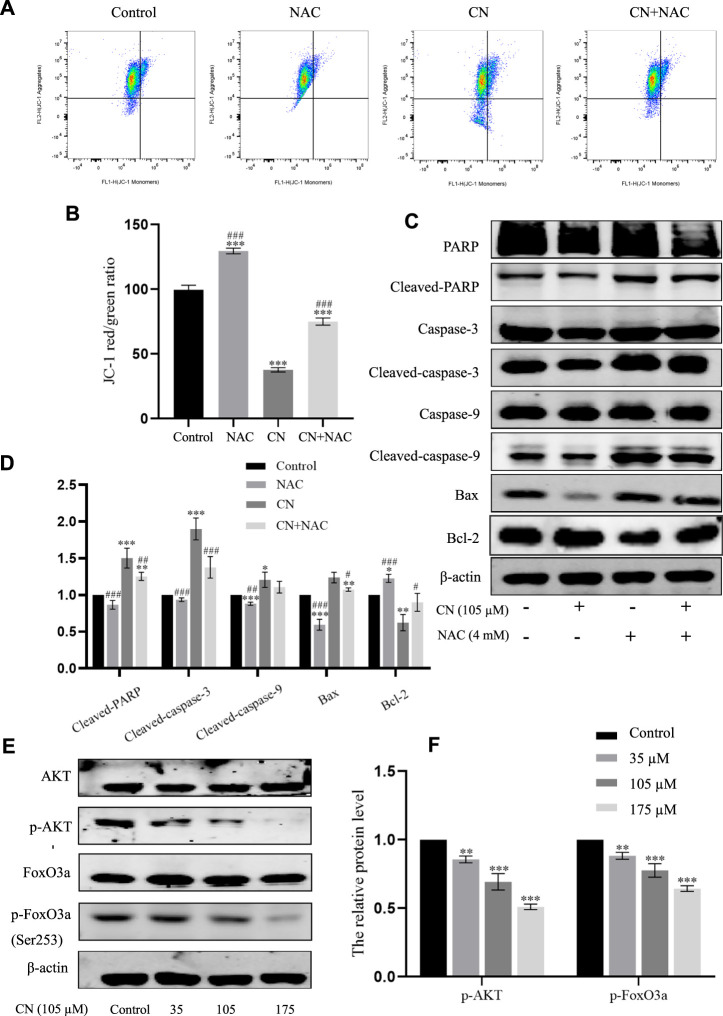
Effects of antioxidant NAC on mitochondria and mitochondria-associated proteins in crebanine-treated HepG2 cells. **(A)** and **(B)** HepG2 cells were pretreated with or without NAC for 1 h before exposure to crebanine, JC-1 staining and flow cytometry were performed to evaluate the effect of crebanine on mitochondrial membrane potential (Δψm) (*n* = 3 for each group, one-way ANOVA with Tukey’s *post hoc* test, ***p < 0.001 vs. Control, ###*p* < 0.001 vs. only crebanine treated groups). **(C)** and **(D)** HepG2 cells were pre-treated with NAC, then treated with crebanine for 24 h. Cleaved PARP, caspase-3, -9, Bcl-2 and Bax levels were determined by Western blotting (*n* = 3 for each group, one-way ANOVA with Tukey’s *post hoc* test, *p < 0.05, **p < 0.01, ***p < 0.001 vs. Control; #*p* < 0.05, ##*p* < 0.01, ###*p* < 0.001 vs. vs. only crebanine treated groups). **(E, F)** Western blot was used to measure the level of signaling pathway proteins (AKT, p-Akt, FoxO3a, p-FoxO3a (Ser253)) (*n* = 3 for each group, one-way ANOVA with Tukey’s *post hoc* test, **p < 0.01, ***p < 0.001 vs. control). All data are expressed as mean ± SD.

Furthermore, to verify whether mitochondrial disorders were due to oxidative stress, we examined mitochondrial membrane potential and the corresponding changes in the expression of these mitochondrial apoptosis-related protein markers after pretreatment with NAC. The results in Figures 6A,B show that NAC delayed the decay of mitochondrial membrane potential and also down-regulated cleaved PARP, cleaved caspase-9, cleaved caspase-3 and Bax, and up-regulated Bcl-2; as seen in Figures 6C,D. These findings provide strong evidence that while inducing ROS, crebanine also triggers mitochondrial dysfunction, further causing cell damage and death.

### Crebanine inhibited the activation of PI3K/AKT/FoxO3a pathway in HepG2 cells

The PI3K/AKT signaling pathway plays a crucial role in the biological functions of growth, proliferation, apoptosis, autophagy, drug resistance, and angiogenesis in various diseases. AKT is a core regulator in the PI3K/AKT signaling pathway, when activated, AKT up-regulates various effector molecules, particularly its downstream target FoxO3a, to promote tumor development. Therefore, in this study, to mechanistically explore the anti-tumor toxicity of crebanine, the effect of crebanine on PI3K/AKT/FoxO3a pathway was examined.

**FIGURE 7:**
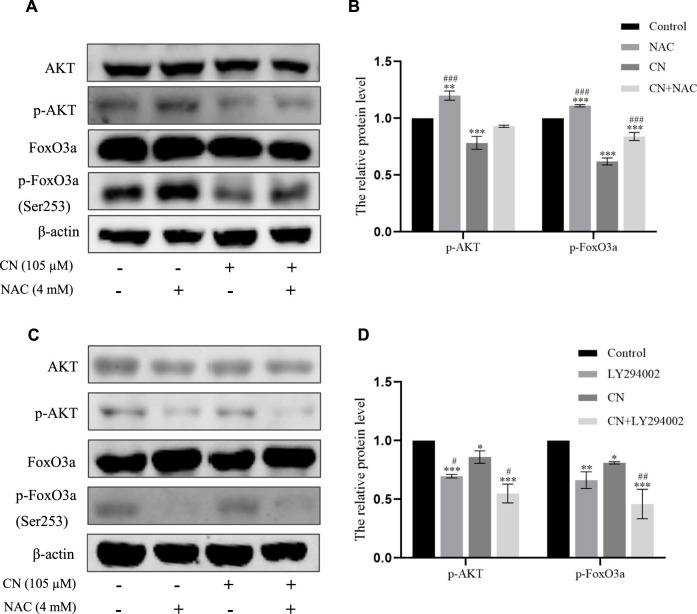
Crebanine induced apoptosis of HepG2 cells through ROS accumulation to regulate the Akt/FoxO3a pathway. **(A)** and **(B)** Cells were pretreated with NAC and then incubated with crebanine for 24 h. Western blot measured the protein levels of AKT, p-Akt, FoxO3a, p-FoxO3a (Ser253) (*n* = 3 for each group, one-way ANOVA with Tukey’s *post hoc* test, **p < 0.01, ***p < 0.001 vs. Control; ###*p* < 0.001 vs. only crebanine treated groups). **(C)** and **(D)** Crebanine was applied in combination with the AKT inhibitor LY294002 to the HepG2 cells to identify the AKT, p-Akt, FoxO3a, p-FoxO3a (Ser253) expression through western blot (n = 3 for each group, one-way ANOVA with Tukey’s *post hoc* test, *p < 0.05, **p < 0.01, ***p < 0.001 vs. Control; #*p* < 0.05 vs. only LY294002 treated groups). All data are expressed as mean ± SD.

The PI3K/AKT signaling pathway plays a crucial role in the biological functions of growth, proliferation, apoptosis, autophagy, drug resistance, and angiogenesis in various diseases. AKT is a core regulator in the PI3K/AKT signaling pathway, when activated, AKT up-regulates various effector molecules, particularly its downstream target FoxO3a, to promote tumor development. Therefore, in this study, to mechanistically explore the anti-tumor toxicity of crebanine, the effect of crebanine on PI3K/AKT/FoxO3a pathway was examined. As shown in Figure 6E, the expression level of AKT remained unchanged after crebanine treatment, whereas that of p-AKT decreased in a dose-dependent way. Although its downstream target FoxO3a was similarly unchanged, AKT inactivation directly down-regulated phosphorylated FoxO3a (Figure 7F).

**FIGURE 8:**
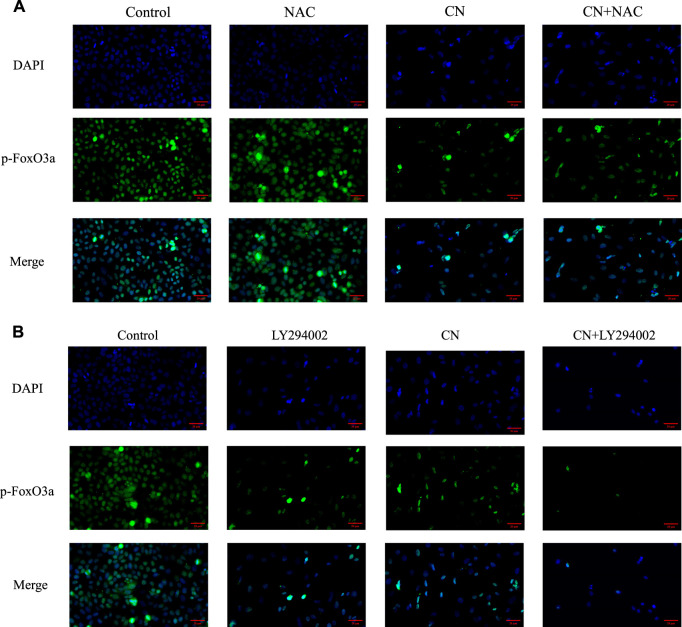
Distribution of p-FoxO3a in HepG2 cells after crebanine action on cells. **(A)** Cells need to be pretreated with NAC before administration of crebanine as a way to monitor changes in p-FoxO3a expression in cells (n = 3 for each group). **(B)** After LY294002 pretreatment of cells, the distribution of p-FoxO3a was detected again (*n* = 3 for each group).

We also pretreated HepG2 cells with NAC for 1 h to examine the impact of ROS generation on the AKT/FoxO3a signaling pathway. The results in Figures 7A,B showed that NAC partially reversed the previous trend of crebanine-induced downregulation of p-AKT and p-FoxO3a (Ser253), whereas AKT and FoxO3a were not significantly changed. This led us to hypothesize that the inhibition of the PI3K/AKT/FoxO3a pathway by crebanine was at least in part caused by the buildup of ROS. In addition, we used immunofluorescence experiments to localize p-FoxO3a (Ser253), and the experimental results shown in Figure 8A showed that p-FoxO3a (Ser253) was indeed more concentrated in the cytoplasm in the control group without the administration of crebanine, and the gradual decrease of p-FoxO3a (Ser253) was indeed inhibited under the conditions of NAC administration, which fully supported our above conclusion.

Besides, LY294002 is a common PI3K/AKT signaling inhibitor. A significant inhibition of AKT and FoxO3a phosphorylation after treatment with a combination of 35 mM LY294002 and crebanine was observed (Figures 6C,D), Similarly, the results of immunofluorescence experiments shown in Figure 8B also suggest that our LY294002 has a significant role in promoting the inhibition of p-FoxO3a (Ser253) cytoplasmic transfer by crebanine, further verifying the relationship between PI3K/AKT/FoxO3a pathway and crebanine-induced cytotoxicity. In conclusion, these results suggested that crebanine-induced cytotoxicity on HepG2 cells could occur through the PI3k/AKT/FoxO3a signaling pathway.

## Discussion

Liver cancer is a highly malignant disease. A history of chronic hepatitis B and C infection, drinking alcohol for a long time, and eating moldy foods are all associated with a high risk of liver cancer (Craig et al., 2020). In addition, as a solid tumor, liver cancer can only be diagnosed at an advanced stage because of its rapid process and high rate of spread. At this stage, most common treatments are no longer effective and more intensive treatments are required. At this point, most common treatments cannot produce the original curative effect, and therefore, patients require more careful and meticulous treatment. At present, effective drug therapies for HCC are limited, and the existing drugs induce various adverse outcomes during treatment (Jensen et al., 2017; Khemlina et al., 2017).

Studies have found that traditional Chinese medicine not only prolong the survival time of patients with hepatocellular carcinoma, but can also improve the prognosis of such patients (Qi et al., 2015; Gao et al., 2016). In recent decades, herbal medicine has received widespread attention as safer and effective alternatives to conventional drugs. As the FDA’s management of research and development of proprietary Chinese medicines becomes increasingly mature, more herbal medicines have been selected for clinical trials (You et al., 2022). With advances in herbal medicine research, our understanding of herbal drugs is likely to grow, contributing to global acceptance and recognition of Chinese herbal medicine as is a valuable source of lead compounds for development of new drugs (Wang et al., 2022). Herbal medicines may not be developed into different pharmaceutical dosage forms for the treatment of various diseases, but their bioactive derivatives can also be used as candidates for the discovery of new drugs (Liu C. et al., 2019).

Crebanine used in this study was extracted from plants of genus Stephania. Crebanine is one of the most abundant aporphine compounds in these herbs (Le et al., 2017). So far, results of preliminary exploration of the properties and structure of crebanine have been reported, including its various activities and medicinal properties of crebanine preparation (Da et al., 2009; Wang et al., 2016). Previous studies have found that crebanine has a certain inhibitory activity against cancer, but these results are inconclusive. In contrast, we discovered that crebanine dramatically reduces the growth of cancer cells, and for the first time, systematically proved that crebanine is a highly active compound with anticancer activity based on its effects on cell morphology, colony formation, migration, invasion, and apoptosis.

Under normal conditions, maintaining a moderate level of ROS in the body is essential for redox homeostasis and normal cell function. However, excessive activation of ROS can overdraw the antioxidant scavenging capacity of the cell itself and initiate the process of abnormal cell death (Zhou et al., 2019; Chen et al., 2020). The most common mechanism of abnormal cell death is occurs in two main ways: death receptor pathway (external) and mitochondrial-mediated pathway (internal) (Liu J. S et al., 2019). In our study, crebanine significantly promoted apoptosis by up-regulating Bax and down-regulating Bcl-2; meanwhile, increasing the shear levels of PARP, caspase-3 and caspase-9 and reducing the mitochondrial membrane potential. This suggests that the apoptosis of hepatoma cells induced by crebanine exposure is mediated by induction of mitochondrial dysfunction. In addition, we also observed, for the first time, that crebanine significantly promoted the production of ROS. Moreover, the accumulation of ROS not only caused apoptosis, but it also directly induced mitochondrial cascade reaction. After treatment with antioxidant NAC, cell death was reversed, part of the mitochondrial membrane potential returned to normal, and down-regulated apoptosis-related proteins Bax, cleaved-caspase-3, cleaved-caspase-9 and cleaved-PARP, and inhibited Bcl2. This finding suggests that crebanine induces oxidative stress and causes mitochondrial dysfunction, which in turn initiates mitochondrial pathway-mediated apoptosis.

We also investigated the connection between crebanine and the PI3K/AKT/FoxO3a signal pathway to better understand the mechanism of crebanine’s anti-tumor toxicity. Our results confirmed that AKT/FoxO3a signal was regulated by crebanine, and the degree of down-regulation of phosphorylated AKT increased with the increase of drug concentration, which decreased phosphorylated FoxO3a expression. This negative change was slowed down after administration of NAC. Figure 7A shows that NAC simultaneously blocked the down-regulation of p-AKT and p-FoxO3a (Ser253) by crebanine; and immunofluorescence experiments also detected aberrant expression of p-FoxO3a due to the presence of crebanine, with pretreatment with NAC leading to a backregulation of p-FoxO3a (Ser253) expression and the opposite outcome occurring with LY294002. These evidences suggest that crebanine-induced ROS and PI3K/AKT/FoxO3a signaling pathways may be related.

It is worth noting that the ROS burst can activate multiple signaling pathways, including the PI3K/AKT pathway, which has been previously demonstrated (Sharma et al., 2011). PI3K/AKT signal has different functions at different stages of carcinogenesis and in the activation and expression of different downstream targets, with PI3K as the key element upstream of this pathway and AKT signal site as the center of the whole cascade reaction (Liu et al., 2009; Das et al., 2016; Yang et al., 2019; Tewari et al., 2022). In the regulation of apoptosis, the main function of AKT is to phosphorylate and inhibit the pro-apoptotic components of the inherent mechanism of cell death in the cytoplasm (Liu et al., 2006; Lin et al., 2021). FoxO3a is one of the key targets of AKT with a significant impact on various signal molecules (Greer and Brunet, 2005; Calnan and Brunet, 2008). Previous studies have found that activation of the PI3K signal cascade in tumor cells causes activated AKT phosphorylation regulators, which make FoxO3a, to interact with 14-3-3 proteins and are transferred to the cytoplasm where they are unable to exert transcriptional activity. Thus, P13K, AKT, and FoxO3a cooperatively regulate the expression of pro-apoptotic genes, cell cycle regulatory genes, and genes controlling cell dynamic balance (Brunet et al., 1999). This indicates that activated AKT not only negatively regulates transcription and triggers FoxO3a phosphorylation and promotes its transportation from the nucleus to the cytoplasm, but it also promotes FoxO3a-mediated gene expression at its downstream targets, affecting cell proliferation, apoptosis, inflammation, oxidative stress, and other activities (Huang and Tindall, 2007; Mogi et al., 2008; Hagenbuchner and Ausserlechner, 2013; Wang X. et al., 2017).

**FIGURE 9:**
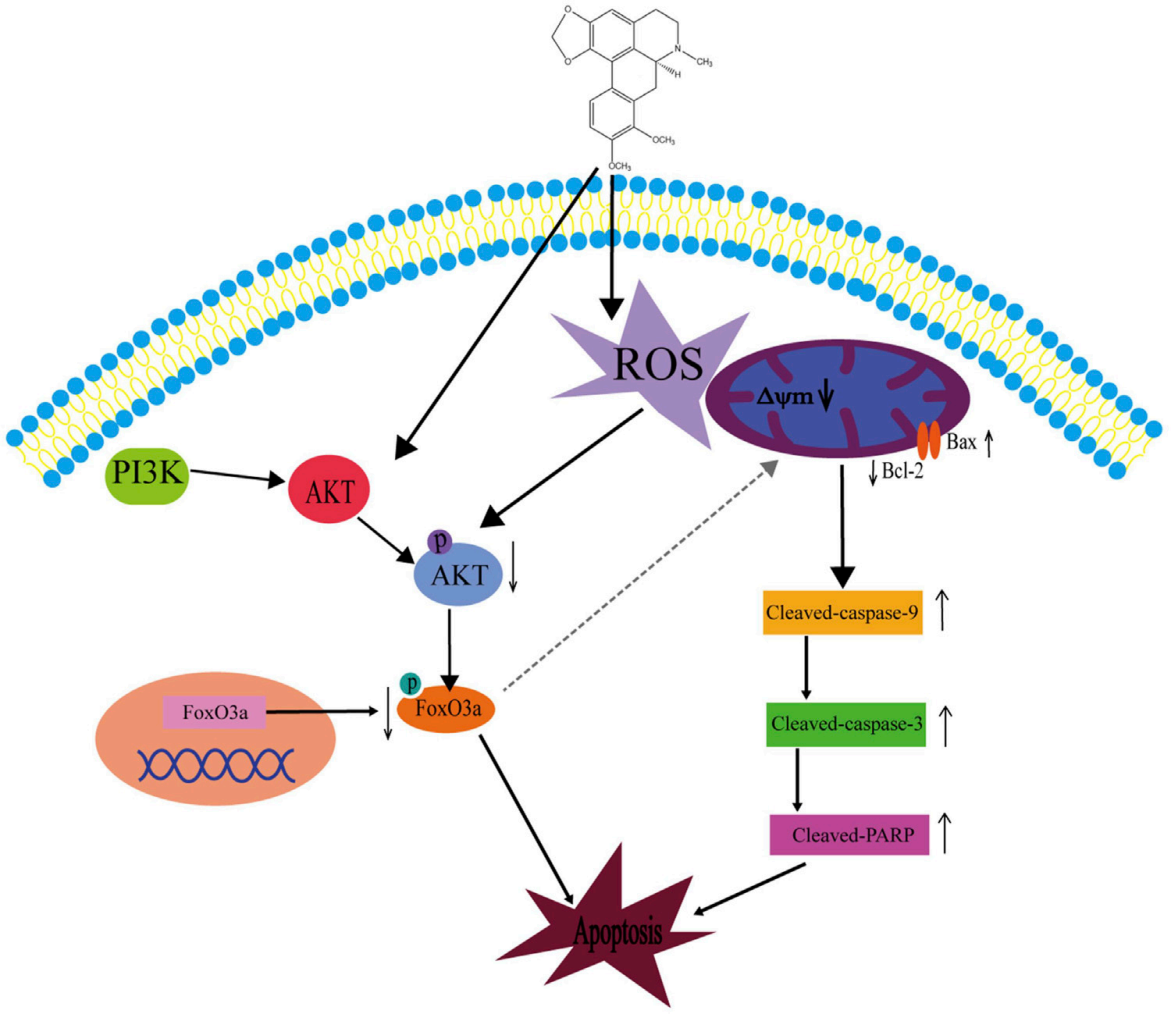
Schematic diagram of the potential signaling pathway triggering apoptosis by crebanine in HepG2 cells. Crebanine treatment triggered the activation of mitochondrial ROS, which caused apoptosis while suppressing the activation of AKT/FoxO3a signaling pathway, further accelerating the death of HepG2 cells.

In summary, we have explored several effects of crebanin on liver cancer. Although we have not carefully studied the relevant mechanism of crebanine inhibition of cell proliferation, migration, and invasion, preliminary experimental results suggest that crebanine has a significant inhibitory function on the occurrence and development of liver cancer. In addition, we thoroughly examined its mode of action on cancer cell apoptosis and discovered that crebanine may controls the transmission of the PI3K/AKT/FoxO3a signal axis to initiate ROS-mediated apoptosis (Figure 9). These findings suggest that crebanine represent a novel therapeutic target for the treatment of cancer.

## Data Availability

The original contributions presented in the study are included in the article/Supplementary Material, further inquiries can be directed to the corresponding author.

## References

[B1] Brunet A. Bonni A. Zigmond M, J. Lin M. Z. Hu. L. S. Arden K. C. (1999). Akt promotes cell survival by phosphorylating and inhibiting a Forkhead transcription factor. Cell 96, 857–868. 10.1016/s0092-8674(00)80595-4 10102273

[B2] Calnan D. R. Brunet A. (2008). The FoxO code. Oncogene 27, 2276–2288. 10.1038/onc.2008.21 18391970

[B3] Chen X. Zhao Y. Luo W. Chen S. Lin F. Zhang X. (2020). Celastrol induces ROS-mediated apoptosis via directly targeting peroxiredoxin-2 in gastric cancer cells. Theranostics 10, 10290–10308. 10.7150/thno.46728 32929349 PMC7481428

[B4] Chiu H. Y. Tay E. X. Y. Ong D. S. T. Taneja R. (2020). Mitochondrial dysfunction at the center of cancer therapy. Antioxid. Redox Signal 32, 309–330. 10.1089/ars.2019.7898 31578870

[B5] Craig A. J. Von F. J. Garcia-Lezana T. Sarcognato S. Villanueva A. (2020). Tumour evolution in hepatocellular carcinoma. Nat. Rev. Gastroenterol. Hepatol. 17, 139–152. 10.1038/s41575-019-0229-4 31792430

[B6] Da S. D. B. Tulli E. C. Militão G. C. Costa-Lotufo L. V. Pessoa C. de Moraes. M. O. (2009). The antitumoral, trypanocidal and antileishmanial activities of extract and alkaloids isolated from Duguetia furfuracea. Phytomedicine 16, 1059–1063. 10.1016/j.phymed.2009.03.019 19423311

[B7] Das T. P. Suman S. Alatassi H. Ankem M. K. Damodaran C. (2016). Inhibition of AKT promotes FoxO3a-dependent apoptosis in prostate cancer. Cell Death Dis. 7, e2111. 10.1038/cddis.2015.403 26913603 PMC4849149

[B8] Fresno V. J. A. Casado E. de C. J. Cejas P. Belda-Iniesta C. González-Barón M. (2004). PI3K/Akt signalling pathway and cancer. Cancer Treat. Rev. 30, 193–204. 10.1016/j.ctrv.2003.07.007 15023437

[B9] Gao L. Wang X. D. Niu Y. Y. Duan D. D. Yang X. Hao J. (2016). Molecular targets of Chinese herbs: A clinical study of hepatoma based on network pharmacology. Sci. Rep. 6, 24944. 10.1038/srep24944 27143508 PMC4855233

[B10] Greer E. L. Brunet A. (2005). FOXO transcription factors at the interface between longevity and tumor suppression. Oncogene 24, 7410–7425. 10.1038/sj.onc.1209086 16288288

[B11] Habrowska-Górczyńska D. E. Kozieł M. J. Kowalska K. Piastowska-Ciesielska A. W. (2021). FoxO3a and its regulators in prostate cancer. Int. J. Mol. Sci. 22, 12530. 10.3390/ijms222212530 34830408 PMC8625444

[B12] Hagenbuchner J. Ausserlechner M.,J. (2013). Mitochondria and FOXO3: Breath or die. Front. Physiol. 4, 147. 10.3389/fphys.2013.00147 23801966 PMC3687139

[B13] Huang H. Tindall D. J. (2007). Dynamic FoxO transcription factors. J. Cell Sci. 120, 2479–2487. 10.1242/jcs.001222 17646672

[B14] Idelchik M. D. P. S. Begley U. Begley T. J. Melendez J. A. (2017). Mitochondrial ROS control of cancer. Semin. Cancer Biol. 47, 57–66. 10.1016/j.semcancer.2017.04.005 28445781 PMC5653465

[B15] Jensen B. C. Parry T. L. Huang W. Beak J. Y. Ilaiwy A. Bain J. R. (2017). Effects of the kinase inhibitor sorafenib on heart, muscle, liver and plasma metabolism *in vivo* using non-targeted metabolomics analysis. Br. J. Pharmacol. 174, 4797–4811. 10.1111/bph.14062 28977680 PMC5727336

[B16] Khemlina G. Ikeda S. Kurzrock R. (2017). The biology of hepatocellular carcinoma: Implications for genomic and immune therapies. Mol. Cancer 16, 149. 10.1186/s12943-017-0712-x 28854942 PMC5577674

[B17] Koundouros N. Poulogiannis G. (2018). Phosphoinositide 3-kinase/akt signaling and redox metabolism in cancer. Front. Oncol. 8, 160. 10.3389/fonc.2018.00160 29868481 PMC5968394

[B18] Kudo M. (2020). Recent advances in systemic therapy for hepatocellular carcinoma in an aging society: 2020 update. Liver Cancer 9, 640–662. 10.1159/000511001 33442538 PMC7768150

[B19] Le P. M. Srivastava V. Nguyen T. T. Pradines B. Madamet M. Mosnier J. (2017). Stephanine from Stephania venosa (blume) spreng showed effective antiplasmodial and anticancer activities, the latter by inducing apoptosis through the reverse of mitotic exit. Phytother. Res. 31, 1357–1368. 10.1002/ptr.5861 28703314

[B20] Li F. Dong X. Lin P. Jiang J. (2018). Regulation of akt/FoxO3a/skp2 Axis is critically involved in berberine-induced cell cycle arrest in hepatocellular carcinoma cells. Int. J. Mol. Sci. 19, 327. 10.3390/ijms19020327 29360760 PMC5855549

[B21] Liang C. Dong Z. Cai X. Shen J. Xu Y. Zhang M. (2020). Hypoxia induces sorafenib resistance mediated by autophagy via activating FoxO3a in hepatocellular carcinoma. Cell Death Dis. 11, 1017. 10.1038/s41419-020-03233-y 33250518 PMC7701149

[B22] Lin R. Liu L. Silva M. Fang J. Zhou Z. Wang H. (2021). Hederagenin protects PC12 cells against corticosterone-induced injury by the activation of the PI3K/AKT pathway. Front. Pharmacol. 12, 712876. 10.3389/fphar.2021.712876 34721013 PMC8551867

[B23] Liu C. Yang S. Wang K. Bao X. Liu Y. Zhou S. (2019). Alkaloids from traditional Chinese medicine against hepatocellular carcinoma. Biomed. Pharmacother. 120, 109543. 10.1016/j.biopha.2019.109543 31655311

[B24] Liu J. S. Huo C. Y. Cao H. H. Fan C. L. Hu J. Y. Deng L. J. (2019). Aloperine induces apoptosis and G2/M cell cycle arrest in hepatocellular carcinoma cells through the PI3K/Akt signaling pathway. Phytomedicine 61, 152843–843. 10.1016/j.phymed.2019.152843 31039533

[B25] Liu L. Z. Hu X. W. Xia C. He J. Zhou Q. Shi X. (2006). Reactive oxygen species regulate epidermal growth factor-induced vascular endothelial growth factor and hypoxia-inducible factor-1alpha expression through activation of AKT and P70S6K1 in human ovarian cancer cells. Free Radic. Biol. Med. 41, 1521–1533. 10.1016/j.freeradbiomed.2006.08.003 17045920

[B26] Liu P. Cheng H. Roberts T. M. Zhao J, J. (2009). Targeting the phosphoinositide 3-kinase pathway in cancer. Nat. Rev. Drug Discov. 8, 627–644. 10.1038/nrd2926 19644473 PMC3142564

[B27] Luo H. Vong C. T. Chen H. Gao Y. Lyu P. Qiu L. (2019). Naturally occurring anti-cancer compounds: Shining from Chinese herbal medicine. Chin. Med. 14, 48. 10.1186/s13020-019-0270-9 31719837 PMC6836491

[B28] Ma Z. Zhang B. Fan Y. Wang M. Kebebe D. Li J. (2019). Traditional Chinese medicine combined with hepatic targeted drug delivery systems: A new strategy for the treatment of liver diseases. Biomed. Pharmacother. 117, 109128. 10.1016/j.biopha.2019.109128 31234023

[B29] Makarasen A. Sirithana W. Mogkhuntod S. Khunnawutmanotham N. Chimnoi N. Techasakul S. (2011). Cytotoxic and antimicrobial activities of apor phine alkaloids isolated from Stephania venosa (Blume) Spreng. PlantaMed 77, 1519–1524. 10.1055/s-0030-1270743 21305448

[B30] Mogi M. Walsh K. Iwai M. Horiuchi M. (2008). Akt-FoxO3a signaling affects human endothelial progenitor cell differentiation. Hypertens. Res. 31, 153–159. 10.1291/hypres.31.153 18360030 PMC5525137

[B31] Moloney J. N. Cotter T. G. (2018). ROS signalling in the biology of cancer. Semin. Cell Dev. Biol. 80, 50–64. 10.1016/j.semcdb.2017.05.023 28587975

[B32] Nogueira V. Park Y. Chen C. C. Xu P. Z. Chen M. L. Tonic I. (2008). Akt determines replicative senescence and oxidative or oncogenic premature senescence and sensitizes cells to oxidative apoptosis. Cancer Cell 14, 458–470. 10.1016/j.ccr.2008.11.003 19061837 PMC3038665

[B33] Patra T. Meyer K. Ray R. B. Kanda T. Ray R. (2021). AKT inhibitor augments anti-proliferative efficacy of a dual mTORC1/2 inhibitor by FoxO3a activation in p53 mutated hepatocarcinoma cells. Cell Death Dis. 12, 1073. 10.1038/s41419-021-04371-7 34759291 PMC8580964

[B34] Plackova P. Sala M. Smidkova M. Dejmek M. Hrebabecky H. Nencka R. (2016). 9-Norbornyl-6-chloropurine (NCP) induces cell death through GSH depletion-associated ER stress and mitochondrial dysfunction. Free Radic. Biol. Med. 97, 223–235. 10.1016/j.freeradbiomed.2016.06.004 27288283

[B35] Qi F. Zhao L. Zhou A. Zhang B. Li A. Wang Z. (2015). The advantages of using traditional Chinese medicine as an adjunctive therapy in the whole course of cancer treatment instead of only terminal stage of cancer. Biosci. Trends 9, 16–34. 10.5582/bst.2015.01019 25787906

[B36] Rojsanga P. Boonyarat C. Utsintong M. Nemecz Á. Yamauchi J. G. Talley T. T. (2012). The effect of crebanine on memory and cognition impairment via the alpha-7 nicotinic acetylcholine receptor. Life Sci. 91, 107–114. 10.1016/j.lfs.2012.06.017 22749860

[B37] Sharma L. K. Fang H. Liu J. Vartak R. Deng J. Bai Y. (2011). Mitochondrial respiratory complex I dysfunction promotes tumorigenesis through ROS alteration and AKT activation. Hum. Mol. Genet. 20, 4605–4616. 10.1093/hmg/ddr395 21890492 PMC3209831

[B38] Sonbol M. B. Riaz I. B. Naqvi S. A. A. Almquist D. R. Mina S. Almasri J. (2020). Systemic therapy and sequencing options in advanced hepatocellular carcinoma: A systematic review and network meta-analysis. JAMA Oncol. 6, e204930. 10.1001/jamaoncol.2020.4930 33090186 PMC7582230

[B39] Sung H. Ferlay J. Siegel R. L. Laversanne M. Soerjomataram I. Jemal A. (2021). Global cancer statistics 2020: GLOBOCAN estimates of incidence and mortality worldwide for 36 cancers in 185 countries. CA Cancer J. Clin. 71, 209–249. 10.3322/caac.21660 33538338

[B40] Tewari D. Patni P. Bishayee A. Sah A. N. Bishayee A. (2022). Natural products targeting the PI3K-Akt-mTOR signaling pathway in cancer: A novel therapeutic strategy. Semin. Cancer Biol. 80, 1–17. 10.1016/j.semcancer.2019.12.008 31866476

[B41] Wang H. Cheng X. Kong S. Yang Z. Wang H. Huang Q. (2016). Synthesis and structure-activity relationships of a series of aporphine derivatives with antiarrhythmic activities and acute toxicity. Molecules 21, 1555. 10.3390/molecules21121555 27916812 PMC6273934

[B42] Wang L. Chen X. Du Z. Li G. Chen M. Chen X. (2017). Curcumin suppresses gastric tumor cell growth via ROS-mediated DNA polymerase γ depletion disrupting cellular bioenergetics. J. Exp. Clin. Cancer Res. 36, 47. 10.1186/s13046-017-0513-5 28359291 PMC5374654

[B43] Wang M. Yao P. F. Sun P. Y. Liang W. Chen X. J. (2022). Key quality factors for Chinese herbal medicines entering the EU market. Chin. Med. 17, 29. 10.1186/s13020-022-00583-x 35193628 PMC8861989

[B44] Wang S. Wu X. Tan M. Gong J. Tan W. Bian B. (2012). Fighting fire with fire: Poisonous Chinese herbal medicine for cancer therapy. J. Ethnopharmacol. 140, 33–45. 10.1016/j.jep.2011.12.041 22265747

[B45] Wang X. Hu S. Liu L. (2017). Phosphorylation and acetylation modifications of FoxO3a: Independently or synergistically? Oncol. Lett. 13, 2867–2872. 10.3892/ol.2017.5851 28521392 PMC5431355

[B46] Wongsirisin P. Yodkeeree S. Pompimon W. Limtrakul P. (2012). Induction of G1 arrest and apoptosis in hum an cancer cells by crebanine,an alkaloid from Stephania venosa. ChemPharmBull (Tokyo) 60, 1283–1289. 10.1248/cpb.c12-00506 22863844

[B47] Xiang Y. Guo Z. Zhu P. Chen J. Huang Y. (2019). Traditional Chinese medicine as a cancer treatment: Modern perspectives of ancient but advanced science. Cancer Med. 8, 1958–1975. 10.1002/cam4.2108 30945475 PMC6536969

[B48] Xiao-Shan H. Qing L. Yun-Shu M. Ze-Pu Y. (2014). Crebanine inhibits voltage-dependent Na^+^ current in Guinea-pig ventricular myocytes. Chin. J. Nat. Med. 12, 20–23. 10.1016/S1875-5364(14)60004-2 24484592

[B49] Yang D. L. Mei W. L. Zeng Y. B. Guo Z. K. Wei D. J. Liu S. B. (2013). A new antibacterial denitroaristolochic acid from the tube rs of Stephania succifera. J. Asian Nat. Prod. Res. 15, 315–318. 10.1080/10286020.2012.762641 23418880

[B50] Yang J. Nie J. Ma X. Wei Y. Peng Y. Wei X. (2019). Targeting PI3K in cancer: Mechanisms and advances in clinical trials. Mol. Cancer 18, 26. 10.1186/s12943-019-0954-x 30782187 PMC6379961

[B51] Yodkeeree S. Pompimon W. Limtrakul P. (2014). Crebanine, an aporphine alkaloid, sensitizes TNF-α-ind uced apoptosis and suppressed invasion of human lung adenocarcinoma cells A549 by blocking NF-κB-regulated gene products. Tumour Biol. 35, 8615–8624. 10.1007/s13277-014-1998-6 24867094

[B52] You L. Liang K. An R. Wang X. (2022). The path towards FDA approval: A challenging journey for traditional Chinese medicine. Pharmacol. Res. 182, 106314. 10.1016/j.phrs.2022.106314 35718244

[B53] Zhou J. Zhang L. Wang M. Zhou L. Feng X. Yu L. (2019). CPX targeting DJ-1 triggers ROS-induced cell death and protective autophagy in colorectal cancer. Theranostics 9, 5577–5594. 10.7150/thno.34663 31534504 PMC6735393

